# *In vivo* morphological alterations of TAMs during KCa3.1 inhibition—by using *in vivo* two-photon time-lapse technology

**DOI:** 10.3389/fncel.2022.1002487

**Published:** 2022-12-15

**Authors:** Francesca Massenzio, Marco Cambiaghi, Federica Marchiotto, Diana Boriero, Cristina Limatola, Giuseppina D’Alessandro, Mario Buffelli

**Affiliations:** ^1^Department of Neurosciences, Biomedicine and Movement Sciences, University of Verona, Verona, Italy; ^2^Department of Physiology and Pharmacology, Sapienza University of Rome, Rome, Italy; ^3^IRCCS Neuromed, Pozzilli, Italy

**Keywords:** Ca^2+^ activated K^+^ channels, TRAM-34, tumor associated macrophages (TAMs), immune cells in the glioma, two-photon imaging

## Abstract

Tumor associated macrophages (TAMs) are the mostprevalent cells recruited in the tumor microenvironment (TME). Once recruited, TAMs acquire a pro-tumor phenotype characterized by a typical morphology: ameboid in the tumor core and with larger soma and thick branches in the tumor periphery. Targeting TAMs by reverting them to an anti-tumor phenotype is a promising strategy for cancer immunotherapy. Taking advantage of Cx3cr1^GFP/WT^ heterozygous mice implanted with murine glioma GL261-RFP cells we investigated the role of Ca^2+^-activated K^+^ channel (KCa3.1) on the phenotypic shift of TAMs at the late stage of glioma growth through *in vivo* two-photon imaging. We demonstrated that TAMs respond promptly to KCa3.1 inhibition using a selective inhibitor of the channel (TRAM-34) in a time-dependent manner by boosting ramified projections attributable to a less hypertrophic phenotype in the tumor core. We also revealed a selective effect of drug treatment by reducing both glioma cells and TAMs in the tumor core with no interference with surrounding cells. Taken together, our data indicate a TRAM-34-dependent progressive morphological transformation of TAMs toward a ramified and anti-tumor phenotype, suggesting that the timing of KCa3.1 inhibition is a key point to allow beneficial effects on TAMs.

## Introduction

Glioblastoma (GBM) is the most common and aggressive brain tumor with a harmful prognosis (Hasselblatt et al., 2018; Tesileanu et al., 2020); according to the World Health Organization, the majority of GBM belong to Grade IV tumor classification with a median survival less than 2 years (Louis et al., 2021). Current clinical treatment for GBM based on resection of the tumor mass, radiotherapy in the tumor area, and concomitant temozolomide chemotherapy is not effective due to the invasiveness, the radio/chemo resistance, and the heterogenic composition of the TME that makes finding a therapeutic approach even more difficult (Weller et al., 2017). TAMs are the most abundant non-neoplastic immune cells in the TME. They consist of both brain-resident microglia and bone-marrow derived macrophages (BMDM), attracted by tumor-released molecules (Buonfiglioli and Hambardzumyan, 2021). Once recruited into the TME, TAMs not only showed a significant expression of anti-inflammatory M2 phenotype markers but were also found to be an important functional component of the TME by supporting tumor progression and glioma resistance to drug treatment (Gabrusiewicz et al., 2011; Li and Graeber, 2012; Wei et al., 2013; Hambardzumyan et al., 2016; Gieryng et al., 2017; Lee et al., 2021). Targeting TAMs to promote their repolarization toward M1-like macrophages resulted in the release of inflammatory factors that support the anti-tumor immune response able to counteract tumor growth and improve the effectiveness of available drugs (Mieczkowski et al., 2015; D’Alessandro et al., 2016; Georgoudaki et al., 2016; Dang et al., 2021; Urbantat et al., 2021). However, there is growing evidence that TAMs activation is dynamic and goes beyond the conventional model of M1/M2 activation, mainly correlating with the phagocytic capacity, thus associated with a hypertrophic morphology of TAMs (Ma et al., 2019; Zhan et al., 2022). In addition, differential TAMs distribution has been reported in a retrospective study showing not only a different distribution between the tumor core and the periphery as a function of TAMs presence but a region-specific morphology of these cells. To note, a ramified morphology was reported in the periphery of the tumor, whereas TAMs into the central region of the tumor were mostly ameboid according to various functional states that reflect the tissue environment (Kvisten et al., 2019). We made use of Cx3cr1^GFP/WT^ heterozygous mice implanted with murine glioma GL261-RFP cells and longitudinal *in vivo* two-photon microscopy to analyze *in vivo* the morphological changes of TAMs and its effect on tumor growth (Madden et al., 2013; Resende et al., 2016).

Our target to modulate TAMs and tumor growth was the Ca^2+^-activated K^+^ channel (KCa3.1). KCa3.1 has been found to be expressed in the brain, primarily by microglial cells in which it regulates migration and proliferation and the immune response to pro-inflammatory stimuli (Kaushal et al., 2007; Maezawa et al., 2011) in different pathological conditions such as Alzheimer’s disease, ischemia and animal model of MS (Reich et al., 2005; Chen et al., 2011; Maezawa et al., 2012). It has been previously shown that KCa3.1 activity is involved in the inflammatory gene expression of glioma infiltrating TAMs and in glioma cell proliferation *in vitro* and *in vivo* (Grimaldi et al., 2016).

In this work, we investigated *in vivo* the effects of KCa3.1 channels inhibition with TRAM-34 1-[(2-chlorophenyl)diphenylmethyl]-1H-pyrazole on the morphological shift of TAMs at the late stage of glioma growth (Wulff et al., 2000; Brown et al., 2018). Taking advantage of two-photon imaging we have constantly monitored TAMs into the tumor mass and recorded responses to channel inhibition. Therefore, we demonstrated that TAMs are highly dynamic cells that promptly respond to KCa3.1 inhibition by reducing their density and the ameboid shape, boosting ramified projections specifically in the tumor core. This trend is in line with the reduced proliferation of glioma cells upon TRAM-34 treatment confirming that affecting TAMs phenotype might be a successful strategy for cancer immunotherapy.

## Materials and Methods

### Cell cultures

Murine glioma GL261-RFP cells, obtained as previously described (Garofalo et al., 2015) were cultured in DMEM supplemented with 20% heat-inactivated FBS, 1% of penicillin/streptomycin (All the reagents are from Invitrogen). Cells were grown at 37°C in a 5% CO_2_ humidified atmosphere and sub-cultivated when confluent.

### Mouse models

Cx3cr1^GFP/WT^ heterozygous mice were housed at a temperature and light-controlled animal facility, at the Interdepartmental Centre for Experimental Research (CIRSAL) of the University of Verona, and received food and water ad libitum. The animal study protocol was approved by the Ethics Committee of the University of Verona and the Italian Ministry of Health (protocol code n° 309/2019- PR, April 2019).

### Mice surgery and TRAM-34 treatment

Eight-week-old Cx3cr1^GFP/WT^ male mice were anesthetized with Ketamine/Xylazine 100 mg/Kg and 10 mg/Kg body weight, respectively and stabilized on a stereotactic frame using ear bars. A hand drill was used to thin the skull and open a cranial window. 1 × 10^5^ GL261-RFP cells in 3 μl PBS were injected 2.0 mm posterior and 1.5 mm lateral of the bregma at 2 mm depth by using a Hamilton syringe (Hamilton) with a 25-gauge needle. A 5 mm-diameter sterile circular cover glass was carefully placed to cover the window and facilitate the *in vivo* imaging procedure, as described in detail in Laperchia et al. (2013). Mice were then housed individually and left to recover for 3 weeks before starting TRAM-34 1-[(2-chlorophenyl) diphenylmethyl]-1H-pyrazole; 120 mg/Kg or vehicle (peanut oil, Sigma Aldrich) intraperitoneal (i.p.) treatment for 10 days. The non-toxicity of the treatment had already been demonstrated (Toyama et al., 2008). See Supplementary Material for additional information and comments on TRAM-34.

### Two-photon imaging acquisition

Taking advantage of cell fluorescence and cranial window surgery to follow the TAMs and glioma cells during tumor growth, two-photon imaging sessions were scheduled to have a baseline acquisition 3 weeks after surgery before starting drug treatment (T0), following one halfway through (T1) and the last one (T2) at the end of TRAM-34 administration. LEICA TCS-SP5 Upright Confocal-Multiphoton Microscope equipped with a long distance 20× water immersion objective was used for live acquisition from the brain surface to a depth of 200 μm. XYZ stack images (1,024 × 1,024 pixels) were taken with a step size of 2.9 μm. XYZT time lapse imaging was performed every 15 min for 1 h.

### Morphological analysis of microglia

XYZ stack images from two-photon acquisition were imported into IMARIS software (BitPlane, Zurich, Switzerland). The morphological analysis of GFP-positive cells (representing the Cx3Cr1 signal) was performed using the Imaris Surface function to measure the area, volume and sphericity of the cell body. The Imaris Filament function was used to measure the cell branching through the quantification of the number of branches, the number of terminal points, and dendrite length (Chen et al., 2019). Microglia branches were considered as “dendrites” by the software. Fifty GFP positive cells from five differently treated or control animals were reconstructed and analyzed for each time point. See Supplementary Material for additional comments.

### Quantitative analysis of microglia and glioma cells in the tumor core

XYZ stack images from two-photon acquisition were imported into ImageJ software. 3D reconstruction was performed using the maximum intensity projection function. Quantitative analysis of TAMs and glioma cells in the tumor core was performed using the Image J plugin Weka Segmentation in order to quantify the % of pixels that were GFP-positive or RFP-positive within a region of interest. Same regions were analyzed to quantify the % of the two cellular populations. See Supplementary Material for additional comments.

### Immunofluorescence staining and quantification

At the end of drug treatment, three mice per group (TRAM-34 or vehicle treated animals) were anesthetized using tribromoethanol (TBE) drug, and perfused transcardially with 0.1 M phosphate buffer solution (Crotty et al., 2008), followed by 40 ml of 4% paraformaldehyde. After the excision, the brain was post-fixed overnight in the same perfusing solution. 40 μm coronal sections were obtained by using the cryostat (Leica, CM1900). Sections with the tumor area were mounted on X-tra adhesive slides (Leica). Once the slices became dry, the slides were immersed in a permeabilization solution, composed of 0.4% Triton-X 100 in PBS with or w/o 2% Goat serum for 30 min at RT. Sections were then treated with rabbit anti-Ki67 (Abcam; 1:500) or anti-CD68 (MCA1957, Bio-Rad; 1:200) in Blocking Solution, composed of 2% Goat Serum, 2% BSA, and 0.4% Triton-X 100 in PBS, for 2 h at RT for Ki67 stain or overnight at 4°C for CD68 stain in dark. After three washes in PBS for 15 min each, sections were incubated with the secondary antibody anti-rabbit CFTM—647 (Merck; 1:1,000) and anti-rat 647 (A21247, Invitrogen; 1:1,000), respectively. Staining with DAPI (1:5,000 in PBS) for 6 min followed by three other PBS washes. Successively, slides were mounted with DAKO Fluorescence mounting medium (DAKO).

### Confocal microscopy acquisition and IMARIS analysis

z-Stacks images (1,024 × 1,024 pixel; step size 0.7 μm) from brain sections were acquired with Leica-Sp5 Confocal Microscope with a 40× oil immersion objective. Eight slices for Ki67 and three slices for CD68 immunofluorescence analysis, per animal, were stained and observed at the microscope. For each slice containing the tumor, three images of the inside, periphery and outside of the tumor were acquired, respectively. The mean number of z-stacks was 3–4 per area. The thickness of each z-stack was 17–20 μm. Three-dimensional image reconstruction was performed with IMARIS software, and two different ROIs were selected randomly for each of the three areas of interest. Ki67 positive cells in the tumor core were manually counted by using the “Manual Edit” option within the “Spot” function of Imaris Software. CD68 signal was analyzed by using the option “Surface” on IMARIS software, allowing a reconstruction of the signal *via* the “Background Subtraction.” Then, the number of voxels occupied by the CD68 signal was divided by the total number of voxels of the ROI.

### Statistical analysis

All data were analyzed by using the GraphPad-Prism8 software and expressed as a mean ± standard error. The two-way analysis of variance was used to compare the means between vehicle and TRAM-34 treated animals.

## Results

### TAMs-glioma model for two-photon *in vivo* imaging

**Figure 1:**
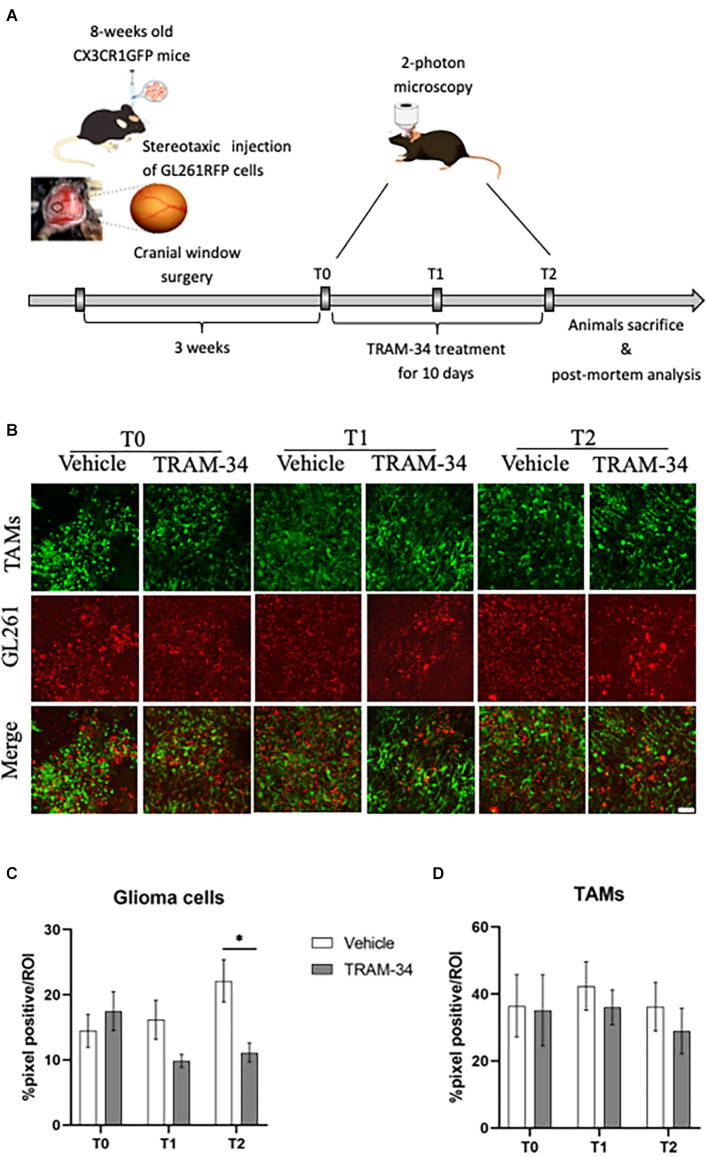
Experimental procedures and analysis of TAMs-glioma interaction *in vivo*. **(A)** Experimental design of drug treatment and two-photon acquisition. **(B)** Representative two-photon images of TAMs-glioma (GL261 cells) interaction before starting drug treatment (T0), halfway through (T1), and at the end (T2) of i.p. treatment with TRAM-34 (120 mg/Kg) or vehicle (100 μl of peanut oil). Scale bar 50 μm. Weka segmentation (ImageJ) quantification of GL261-positive pixel **(C)** or TAMs-positive pixel **(D)** within a picture’s ROI at each day of acquisition (T0, T1, T2) in treated and control mice. Vehicle 10 animals, TRAM-34 nine animals, one picture per animal. TAMs, Tumor associated macrophages. Two-way ANOVA ± S.E. ^*^p < 0.05.

To evaluate the role of KCa3.1 activity in glioma-associated TAMs morphology, we took advantage of Cx3cr1^GFP/WT^ heterozygous mice intracortical implanted with GL261-RFP cells. In this mouse model of TAMs-glioma interaction, we were able, by two-photon imaging to follow the TAMs (GFP) and tumor cells (RFP) during the tumor growth. It has been already demonstrated that Cx3cr1 deletion or replacement with GFP did not affect TAMs infiltration in tumor bearing mice (Liu et al., 2008; Feng et al., 2015). All image stacks were acquired up to a depth of ~200 μm from the surface and the maximum projection of a 3D confirmed the good quality of the fluorescence intensity until the end of the experimental procedure. First, we verified that the whole experimental procedure did not affect animal health (Supplementary Figure S1), as already proved by Toyama and colleagues (Toyama et al., 2008). To evaluate the role of KCa3.1 in TAMs we pharmacologically inhibited the channel expressed in both glioma and immune cells (Fioretti et al., 2006; Weaver et al., 2006; Nguyen et al., 2017). For this purpose, mice were treated with TRAM-34 starting 3 weeks after the surgery to reduce the inflammation of the tissue and the activation of microglia (Wulff et al., 2000). From this time point, mice were daily administered with TRAM-34 (120 mg/kg) or peanut oil for 10 days and *in vivo* acquisition was performed every 5 days as shown in Figure 1A.

### KCa3.1 channel inhibition reduces the proliferation of glioma cells

Previous works showed that the mean survival time of GL261glioma-bearing mice was of about 30 days after tumor cell inoculation (Pellegatta et al., 2006; D’Alessandro et al., 2016). In order to follow TAMs morphology and the role of KCa3.1 channels at the most acute stage of tumor growth and invasiveness, GL261 bearing mice were evaluated by *in vivo* two-photon imaging at 21 (T0), 26 (T1), and 31 (T2) days after tumor cell inoculation. At T0, the chronic inhibition of KCa3.1 was started by daily administration of TRAM-34 (Figure 1A). Figure 1B shows representative images of two-photon acquisition at the set time points of treatment. Green, fluorescent TAMs infiltrated the total tumor mass and exhibited a typical amoeboid shape in the tumor core (Supplementary Figure S2) clearly observable at T0 acquisition and preserved in vehicle-treated mice. KCa3.1 channel inhibition reduced the proliferation of glioma cells over time from 5 to 10 days of treatment with a significant decrease compared to vehicle-treated mice (Figure 1C). TAMs recruitment into the tumor mass was also quantified. As observed in Figure 1D, there were no significant changes in the quantity of TAMs over time although a slight decrease of GFP-positive cells was measured in TRAM-34-treated mice at the end point.

**Figure 2:**
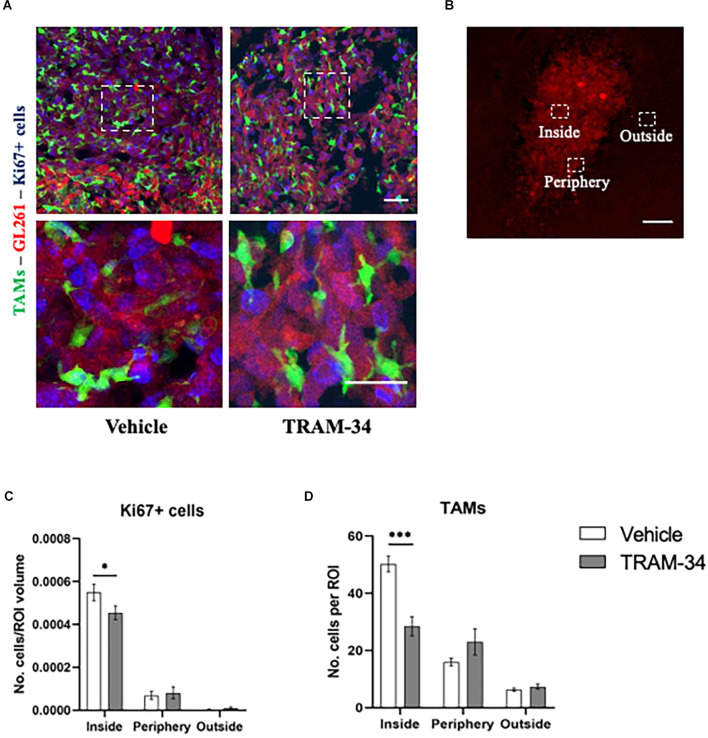
Immunofluorescence analysis of proliferation marker Ki67. **(A)** Representative confocal images at 40× magnification of inside the tumor of CX3CR1 mice, stained with anti-Ki67 antibody (blu) with a higher magnification below, at T2. Scale bar = 30 μm. **(B)** Representative 2D image of the tumor cells in a brain slice of 40 μm. The boxes indicate the three considered areas. The image was acquired with a fluorescence microscope, 5× objective. Scale bar = 100 μm. **(C)** The number of Ki67 positive cells per region of interest (ROI) and **(D)** number of TAMs per ROI inside, peripherical, and outside the tumor of vehicles and TRAM-34 groups (*n* = 3/group). Analysis with two-way ANOVA, mean ± SEM ^*^p < 0.05; ^***^p < 0.001.

To further confirm the power of TRAM-34 in reducing cell proliferation, we stained coronal slice and quantified the number of Ki67 positive cells at the end point of the treatment (T2). Representative Figure 2A shows Ki67 stain and GFP-positive cells in the tumoral mass of vehicle and TRAM-34 treated mice. Quantification of Ki67 positive cells was performed in the core, the periphery and outside the tumor mass separately (Figure 2B). As shown in Figure 2C, we found a significant reduction of Ki67-positive cells inside the tumor after 10 days of TRAM-34 administration (T2) compared to vehicle-treated mice. Moving out (periphery and outside tumor mass), no differences in Ki67 quantification were reported (Figure 2C). The same ROIs were also evaluated for the presence of TAMs recruited into the TME. GFP-positive cells counting in Figure 2D, showed a significant reduction between vehicle and TRAM-34 treated mice in line with the reduction of Ki67 suggesting that the recruitment of TAMs, as well as tumor cell proliferation, were affected by the treatment only in the tumor core region.

### KCa3.1 channel inhibition promotes morphological changes of TAMs in the tumor microenvironment

**Figure 3:**
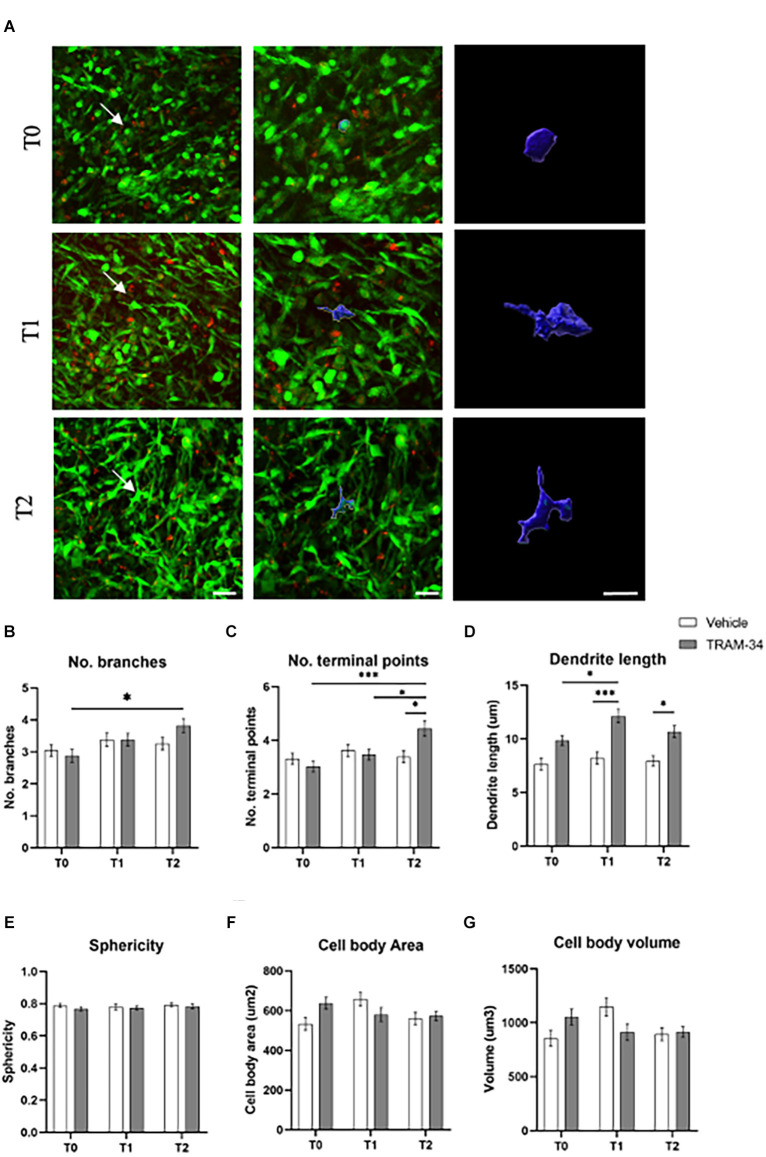
Morphology analysis of TAMs. **(A)** Representative 3D IMARIS reconstruction of TAMs. The reconstruction above was performed at T0, T1, and T2 of TRAM-34 treatment. The images on the center (scale bar = 20 μm) and in the right (scale bar = 10 μm) show the reconstruction of TAMs indicated with a white arrow in the picture on the left (scale bar = 30 μm). **(B)** The number of branches/cell. **(C)** The number of terminal points/cells. **(D)** The total branch length for each cell is considered as “dendrite” for the IMARIS Filaments function (μm). **(E)** Sphericity (1.0 perfect sphere; 0 non sphere). **(F)** Cell area (μm^2^). **(G)** Cell volume (μm^3^). White bars are for vehicle-treated animals, while the gray ones are for TRAM-34-treated animals. 50 cells from five mice. Two-way ANOVA followed by Tukey’s *Post hoc* correction ± S.E. ^*^p < 0.05; ^***^p < 0.001. Scale bar 10 μm.

Two-photon XYZ stacks acquisitions at each time point for TRAM-34- or vehicle-treated animals were imported into IMARIS for the creation of 3D images (Figure 3A and Supplementary Figure S3). TAMs cells were identified according to the green fluorescence in the tumor mass. Ten cells per image, of five animals for each experimental group were randomly analyzed before the treatment (T0), halfway through the treatment (T1), and at the end point (T2). The most significant differences in cell morphology were related to the branches’ conformation. An increased number of branches was measured for TRAM-34-treated animals over time (Figure 3B), together with the increase in the number of terminal points (Figure 3C) and the length of ramification (Figure 3D) attributable to an activated non-ameboid phenotype. Looking at the cells in control animals, the morphology of the ramification has not changed over the 10 days of treatment confirming the role of the KCa3.1 channel in modulating the morphology of TAMs. The mean surface area, volume, and sphericity (Figures 3E–G) were not changed by TRAM-34 treatment compared to control animals.

XYZT time-lapse images were then analyzed by using the Image J plug in TrackMate to define the migratory ability of TAMs into the tumor microenvironment as a function of the morphological changes promoted by drug treatment. TAMs speed within the tumor core was measured by following cells selected based on threshold and size within an ROI from time 0 up to 60 min (Supplementary Figure S4A). Microglia activation depends on different stimuli and its migratory activity seems to be boosted in the alternative-activated cells as well as KCa3.1 blocking inhibits chemotactic migration *in vitro* (Ferreira and Schlichter, 2013; Lively and Schlichter, 2013; Ferreira et al., 2014). In this regard, TAMs from TRAM-34-treated mice showed a slight reduction in migratory speed after 5 days of treatment (T1) even though the end point was comparable to the baseline level, emphasizing once again the complex behavior of TAMs in response to activating stimuli (Supplementary Figure S4B).

### KCa3.1 channel inhibition promotes the reduction of phagocytic activity of TAMs in the tumor microenvironment

**Figure 4:**
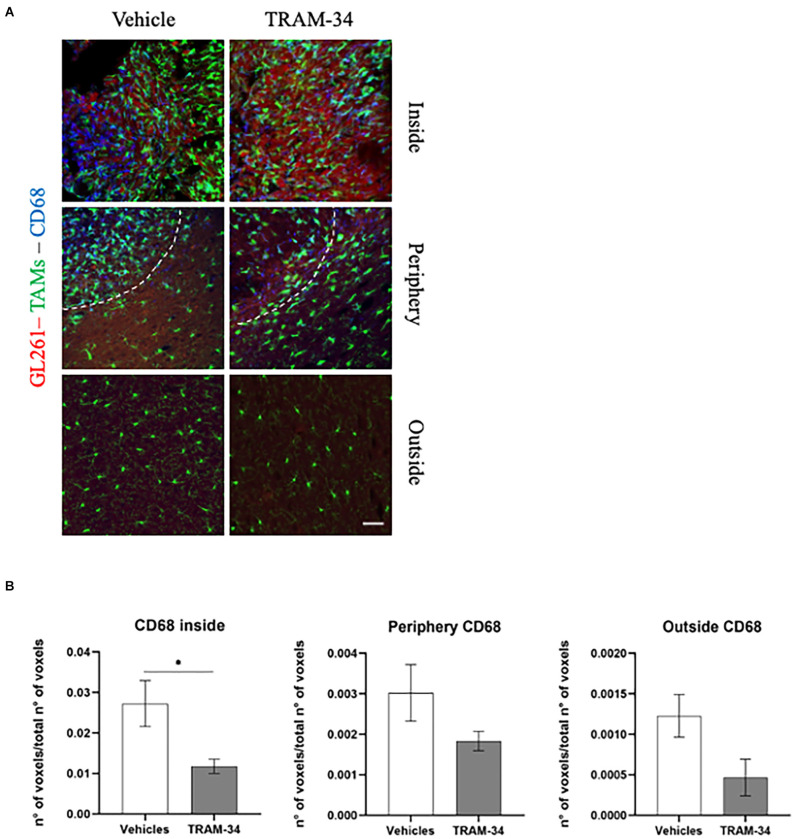
Immunofluorescence analysis of phagocytic marker CD68. **(A)** Representative confocal images at 40× magnification of the tumor of CX3CR1 mice, stained with anti-CD68 antibody (blue), at T2. Scale bar = 30 μm. **(B)** Number of CD68-positive voxels over the total number of voxels per ROIs inside, peripherical and outside the tumor of vehicles and TRAM-34 groups (*n* = 4/group). Analysis with unpaired t-test; mean ± SEM, ^*^p < 0.05.

The cluster of differentiation CD68, overexpressed in TAMs, not only indicates the amoeboid, phagocytic phenotype of those cells (da Silva and Gordon, 1999) but it is also related to a higher-grade tumor, Ki67 positivity, and tumor aggressiveness (Sun et al., 2016; Zhu et al., 2016; Ni et al., 2019). In this regard, to better outline the functional profile of TAMs in our model, we stained the coronal slice and quantified the expression of CD68 at the end point of TRAM-34 treatment (T2). Representative Figure 4A shows CD68-positive cells and TAMs inside, in the periphery, and outside the tumor mass of vehicle and TRAM-34 treated mice. As shown in Figure 4B, we found a significant reduction of CD68 expression inside the tumor after 10 days of TRAM-34 administration compared to vehicle-treated mice. A similar trend was identified even in the periphery, and outside the tumor suggesting that the phagocytic activation was reduced in mice treated with TRAM-34 as well as tumor progression (Sun et al., 2016; Zhu et al., 2016; Ni et al., 2019).

## Discussion

*In vivo* models of glioma are becoming a widely used model to study TAMs biology and their role in cancer immunotherapy (Resende et al., 2016; Gieryng et al., 2017; Chen et al., 2019; Dugandžija et al., 2021; Zhang et al., 2021). Cx3cr1^GFP/WT^—GL261-RFP model, together with cranial window surgery, gives the opportunity to longitudinal follow the morphometric properties of TAMs during tumor growth.

We decided to start TRAM-34 treatment and imaging acquisition 3 weeks after tumor implantation for three main reasons: (i) let the cranial window become clear to ensure good image quality; (ii) have a reliable pro-tumorigenic phenotype of TAMs to test the effectiveness of KCa3.1 inhibition in the modulation of TAMs morphology and therefore their immunomodulatory functions (Gabrusiewicz et al., 2011; Gieryng et al., 2017; Dugandžija et al., 2021); and (iii) test the effectiveness of KCa3.1 inhibition at the end stage of tumor growth in order to define the better timing for drug treatment in comparison with previous studies that investigated longer treatment and at an early stage of tumor progression.

KCa3.1 channel is overexpressed in 32% of glioma patients and correlates with significantly shortened survival (Turner et al., 2014). In glioma cell lines KCa3.1 expression level correlates with cell invasiveness in response to the chemokine CXCL12, serum, and bradykinin (Sciaccaluga et al., 2010; Catacuzzeno et al., 2011; Cuddapah et al., 2013). As already demonstrated, KCa3.1 inhibition affects microglia proliferation, p38 MAPK phosphorylation, NF-kβ activation, and NO production (Kaushal et al., 2007; Maezawa et al., 2011) as well as the migration properties through the FAK (focal adhesion kinase) and PI3K/AKT pathway signaling downstream KCa3.1 activation (Giannone et al., 2002; Ferreira and Schlichter, 2013).

We demonstrated that KCa3.1 channel inhibition was able to decrease glioma cell proliferation. This result is in line with the reduced infiltrative behavior of human glioma cells silenced for KCa3.1 and implanted in SCID mice (D’Alessandro et al., 2013; Turner et al., 2014). In addition, we also showed a reduction of TAMs density inside the tumor area, suggesting a reduced recruitment of TAMs in the TME. TAMs respond to KCa3.1 inhibition *via* morphological changes (Hutter et al., 2019). TAMs underwent morphological changes once in contact with glioma cells as described in human glioma (Kvisten et al., 2019) and in glioma-bearing animal models (Fernández-Arjona et al., 2017; Sørensen et al., 2018; Dugandžija et al., 2021). TAMs contacted GL261-RFP cells in Cx3cr1^GFP/WT^ mice shortly after tumor injection turning toward an ameboid shape characterized by larger soma and shortened, thick branches found in the tumor core (Resende et al., 2016; Fernández-Arjona et al., 2017; Sørensen et al., 2018; Kvisten et al., 2019; Dugandžija et al., 2021) that correlates with a significant increase of M2-phenotype markers and reduced expression of inflammation mediators (Gieryng et al., 2017). Ameboid TAMs were found in the tumor area at the first acquisition (T0) in agreement with the activated state demonstrated for infiltrating microglia in glioma-bearing models (Gabrusiewicz et al., 2011; Gieryng et al., 2017). Daily administration of TRAM-34 promoted the morphological change of microglia toward a more complex and ramified shape attributable to an activated non-ameboid phenotype characterized by branched cells with highly active processes (Chen et al., 2019; Qiao et al., 2019; Zhang et al., 2021). By exploiting two-photon imaging we demonstrated for the first time that five days of treatment with KCa3.1 inhibitor was enough to promote a partial morphological change in TAMs that became more significant at the end point. Our results are supported by a recent study that characterized the different activation states of TAMs according to the branching parameters *in vivo* (Qiao et al., 2019). Despite this, we failed to detect an increase in body area and volume associated with pro-tumor polarization of TAMs probably due to the timing of our experimental procedures which aimed to inhibit KCa3.1 channels at an advanced stage of glioblastoma growth. Nevertheless, we demonstrated that 10 days of TRAM-34 administration were enough to reduce cell proliferation inside the tumor without affecting the periphery proving a specific effect of drug treatment in the tumor core with no interference with surrounding cells.

Further, we demonstrated that ameboid TAMs and increased cell proliferation within the tumor are associated with CD68 expression. The reduced expression of CD68, a marker of phagocytic macrophages (da Silva and Gordon, 1999) inside the tumor, correlates with the reduction of the ameboid morphology of TAMs promoted by KCa3.1 channels inhibition, allowing us to also validate the functional properties of TAMs in the TME in response to TRAM-34. Thus, the modulation of CD68 expression in accordance with the reduction of cell proliferation measured within the tumor confirms the ability of TRAM-34 to reduce glioma invasiveness since the overexpression of CD68, recently found in several types of cancer, has been correlated with tumor aggressiveness and malignant progression (Zhang et al., 2022).

The anti-tumor efficacy of TRAM-34 in terms of reduction of migration, invasiveness, and phagocytosis of TAMs depends on FAK and AKT phosphorylation (Grimaldi et al., 2016) as well as tumor infiltration in the brain parenchyma (D’Alessandro et al., 2013). Also, Ca^2+^ influx regulated by K^+^ channels works as a temporal and spatial regulator of cell migration coordinating the retraction of the rear part of migrating cells with forward protrusion (Duffy et al., 2007; Fabian et al., 2008). Blocking the channels with TRAM-34 resulted in a slight reduction of migratory speed of TAMs in time-lapse acquisition at T1 compared to T0 as well as compared with the control group at the same day time. This result overlapped the inhibition of chemotactic activity reported in response to glioblastoma-conditioned medium (D’Alessandro et al., 2013) or IL-4 (Ferreira et al., 2014). Nevertheless, the migratory capacity of TAMs in our model is intriguing. See Supplementary Material for additional comments.

A detailed analysis in terms of the migratory capacity of brain resident microglia BMDM showed no migration property for microglial cells, mainly found at the periphery of the tumor margins while infiltrating inflammatory monocytes, found both in the tumor bulk and in the tumor margins were found to be migratory (Chen et al., 2019).

Also, it has been reported that blocking KCa3.1 with TRAM-34 inhibited chemotactic migration of rat microglia since K^+^ efflux through the channel followed by Ca^2+^ influx due to the electrochemical gradient helps the cells to modify their shape promoting the migration (Ferreira et al., 2014).

Considering this evidence, we can speculate that the slightly reduced migratory capacity of TAMs at day 5 of treatment with TRAM-34 depends on the electrochemical gradient modified by the inhibition of KCa3.1 on recruited cells within the tumor. In addition, the heterogeneity of TAMs population, might be the limiting factor that does not allow us to clearly define the migratory properties of microglia and macrophages.

In conclusion, we can hypothesize a progressive and non-complete polarization of TAMs from the pro-tumor phenotype toward the anti-tumor one suggesting that the timing of KCa3.1 inhibition is a key point to allow beneficial effects on TAMs.

## Data Availability Statement

The raw data supporting the conclusions of this article will be made available by the authors, without undue reservation.

## Ethics Statement

The animal study was reviewed and approved by the Ethics Committee of the University of Verona and the Italian Ministry of Health (protocol code n° 309/2019- PR, Apri 2019).

## Author Contributions

MB and CL: conceptualization and funding acquisition. FMas, MC, DB, and FMar: methodology and data curation. FMas and MB: writing—original draft preparation. FMas, GD’A, CL, and MB: writing—review and editing. MB: supervision. All authors contributed to the article and approved the submitted version.

## Conflict of Interest

The authors declare that the research was conducted in the absence of any commercial or financial relationships that could be construed as a potential conflict of interest.

## Publisher’s Note

All claims expressed in this article are solely those of the authors and do not necessarily represent those of their affiliated organizations, or those of the publisher, the editors and the reviewers. Any product that may be evaluated in this article, or claim that may be made by its manufacturer, is not guaranteed or endorsed by the publisher.

## References

[B1] Brown B. M. Pressley B. Wulff H. (2018). KCa3.1 channel modulators as potential therapeutic compounds for glioblastoma. Curr. Neuropharmacol. 16, 618–626. 10.2174/1570159X15666170630164226 28676010 PMC5997873

[B2] Buonfiglioli A. Hambardzumyan D. (2021). Macrophages and microglia: the cerberus of glioblastoma. Acta Neuropathol. Commun. 9:54. 10.1186/s40478-021-01156-z 33766119 PMC7992800

[B3] Catacuzzeno L. Aiello F. Fioretti B. Sforna L. Castigli E. Ruggieri P.. (2011). Serum-activated K and Cl currents underlay U87-MG glioblastoma cell migration. J. Cell. Physiol. 226, 1926–1933. 10.1002/jcp.22523 21506123

[B4] Chen Y. J. Raman G. Bodendiek S. O’Donnell M. E. Wulff H. (2011). The KCa3.1 blocker TRAM-34 reduces infarction and neurological deficit in a rat model of ischemia/reperfusion stroke. J. Cereb. Blood Flow Metab. 31, 2363–2374. 10.1038/jcbfm.2011.101 21750563 PMC3323185

[B6] Chen Z. Ross J. L. Hambardzumyan D. (2019). Intravital 2-photon imaging reveals distinct morphology and infiltrative properties of glioblastoma-associated macrophages. Proc. Natl. Acad. Sci. U S A 116, 14254–14259. 10.1073/pnas.1902366116 31235603 PMC6628659

[B7] Crotty S. Fitzgerald P. Tuohy E. Harris D. M. Fisher A. Mandel A.. (2008). Neuroprotective effects of novel phosphatidylglycerol-based phospholipids in the 6-hydroxydopamine model of Parkinson’s disease. Eur. J. Neurosci. 27, 294–300. 10.1111/j.1460-9568.2007.06018.x 18190522

[B8] Cuddapah V. A. Turner K. L. Seifert S. Sontheimer H. (2013). Bradykinin-induced chemotaxis of human gliomas requires the activation of KCa3.1 and ClC-3. J. Neurosci. 33, 1427–1440. 10.1523/JNEUROSCI.3980-12.2013 23345219 PMC3711544

[B9] da Silva R. P. Gordon S. (1999). Phagocytosis stimulates alternative glycosylation of macrosialin (mouse CD68), a macrophage-specific endosomal prote. Biochem. J. 338, 687–694. 10051440 PMC1220104

[B10] D’Alessandro G. Catalano M. Sciaccaluga M. Chece G. Cipriani R. Rosito M.. (2013). KCa3.1 channels are involved in the infiltrative behavior of glioblastoma *in vivo*. Cell Death Dis. 4:e773. 10.1038/cddis.2013.279 23949222 PMC3763441

[B11] D’Alessandro G. Grimaldi A. Chece G. Porzia A. Esposito V. Santoro A.. (2016). KCa3.1 channel inhibition sensitizes malignant gliomas to temozolomide treatment. Oncotarget 7, 30781–30796. 10.18632/oncotarget.8761 27096953 PMC5058717

[B12] Dang W. Xiao J. Ma Q. Miao J. Cao M. Chen L.. (2021). Combination of p38 MAPK inhibitor with PD-L1 antibody effectively prolongs survivals of temozolomide-resistant glioma-bearing mice via reduction of infiltrating glioma-associated macrophages and PD-L1 expression on resident glioma-associated microglia. Brain Tumor Pathol. 38, 189–200. 10.1007/s10014-021-00404-3 34231121

[B13] Duffy S. M. Cruse G. Brightling C. E. Bradding P. (2007). Adenosine closes the K^+^ channel KCa3.1 in human lung mast cells and inhibits their migration via the adenosine A2A receptor. Eur. J. Immunol. 37, 1653–1662. 10.1002/eji.200637024 17474152 PMC2699420

[B14] Dugandžija T. Drljača J. Bulajić D. Isaković A. Stilinović N. Sekulić S.. (2021). Hallmarks of tumor-associated microglia response to experimental U87 human glioblastoma xenograft. Tissue Cell 72:101557. 10.1016/j.tice.2021.101557 34051646

[B15] Fabian A. Fortmann T. Dieterich P. Riethmüller C. Schön P. Mally S.. (2008). TRPC1 channels regulate directionality of migrating cells. Pflugers Arch. 457, 475–484. 10.1007/s00424-008-0515-4 18542994

[B16] Feng X. Szulzewsky F. Yerevanian A. Chen Z. Heinzmann D. Rasmussen R. D.. (2015). Loss of CX3CR1 increases accumulation of inflammatory monocytes and promotes gliomagenesis. Oncotarget 6, 15077–15094. 10.18632/oncotarget.3730 25987130 PMC4558137

[B17] Fernández-Arjona M. D. M. Grondona J. M. Granados-Durán P. Fernández-Llebrez P. López-Ávalos M. D. (2017). Microglia morphological categorization in a rat model of neuroinflammation by hierarchical cluster and principal components analysis. Front. Cell. Neurosci. 11:235. 10.3389/fncel.2017.00235 28848398 PMC5550745

[B19] Ferreira R. Lively S. Schlichter L. C. (2014). IL-4 type 1 receptor signaling up-regulates KCNN4 expression,and increases the KCa3.1 current and its contribution to migration of alternative-activated microglia. Front. Cell. Neurosci. 8:183. 10.3389/fncel.2014.00183 25071444 PMC4077126

[B18] Ferreira R. Schlichter L. C. (2013). Selective activation of KCa3.1 and CRAC channels by P2Y2 receptors promotes Ca^2+^ signaling, store refilling and migration of rat microglial cells. PLoS One 8:e62345. 10.1371/journal.pone.0062345 23620825 PMC3631179

[B20] Fioretti B. Castigli E. Micheli M. R. Bova R. Sciaccaluga M. Harper A.. (2006). Expression and modulation of the intermediate- conductance Ca^2+^-activated K^+^ channel in glioblastoma GL-15 cells. Cell. Physiol. Biochem. 18, 47–56. 10.1159/000095135 16914889

[B22] Gabrusiewicz K. Ellert-Miklaszewska A. Lipko M. Sielska M. Frankowska M. Kaminska B. (2011). Characteristics of the alternative phenotype of microglia/macrophages and its modulation in experimental gliomas. PLoS One 6:e23902. 10.1371/journal.pone.0023902 21901144 PMC3162015

[B23] Garofalo S. D’Alessandro G. Chece G. Brau F. Maggi L. Rosa A.. (2015). Enriched environment reduces glioma growth through immune and non-immune mechanisms in mice. Nat. Commun. 6:6623. 10.1038/ncomms7623 25818172 PMC4389244

[B24] Georgoudaki A. M. Prokopec K. E. Boura V. F. Hellqvist E. Sohn S. Östling J.. (2016). Reprogramming tumor-associated macrophages by antibody targeting inhibits cancer progression and metastasis. Cell Rep. 15, 2000–2011. 10.1016/j.celrep.2016.04.084 27210762

[B25] Giannone G. Rondé P. Gaire M. Haiech J. Takeda K. (2002). Calcium oscillations trigger focal adhesion disassembly in human U87 astrocytoma cells. J. Biol. Chem. 277, 26364–26371. 10.1074/jbc.M203952200 12011063

[B26] Gieryng A. Pszczolkowska D. Bocian K. Dabrowski M. Rajan W. D. Kloss M.. (2017). Immune microenvironment of experimental rat C6 gliomas resembles human glioblastomas. Sci. Rep. 7:17556. 10.1038/s41598-017-17752-w 29242629 PMC5730558

[B27] Grimaldi A. D’Alessandro G. Golia M. T. Grössinger E. M. Di Angelantonio S. Ragozzino D.. (2016). KCa3.1 inhibition switches the phenotype of glioma-infiltrating microglia/macrophages. Cell Death Dis. 7:e2174. 10.1038/cddis.2016.73 27054329 PMC4855657

[B28] Hambardzumyan D. Gutmann D. H. Kettenmann H. (2016). The role of microglia and macrophages in glioma maintenance and progression. Nat. Neurosci. 19, 20–27. 10.1038/nn.4185 26713745 PMC4876023

[B29] Hasselblatt M. Jaber M. Reuss D. Grauer O. Bibo A. Terwey S.. (2018). Diffuse astrocytoma, IDH-wildtype: a dissolving diagnosis. J. Neuropathol. Exp. Neurol. 77, 422–425. 10.1093/jnen/nly012 29444314

[B30] Hutter G. Theruvath J. Graef C. M. Zhang M. Schoen M. K. Manz E. M.. (2019). Microglia are effector cells of CD47-SIRPα antiphagocytic axis disruption against glioblastoma. Proc. Natl. Acad. Sci. U S A 116, 997–1006. 10.1073/pnas.1721434116 30602457 PMC6338872

[B31] Kaushal V. Koeberle P. D. Wang Y. Schlichter L. C. (2007). The Ca^2+^-activated K^+^ channel KCNN4/KCa3.1 contributes to microglia activation and nitric oxide-dependent neurodegeneration. J. Neurosci. 27, 234–244. 10.1523/JNEUROSCI.3593-06.2007 17202491 PMC6672279

[B32] Kvisten M. Mikkelsen V. E. Stensjøen A. L. Solheim O. Van Der Want J. Torp S. H. (2019). Microglia and macrophages in human glioblastomas: a morphological and immunohistochemical study. Mol. Clin. Oncol. 11, 31–36. 10.3892/mco.2019.1856 31289674 PMC6535640

[B33] Laperchia C. Allegra Mascaro A. L. Sacconi L. Andrioli A. Mattè A. De Franceschi L.. (2013). Two-photon microscopy imaging of thy1GFP-M transgenic mice: a novel animal model to investigate brain dendritic cell subsets in vivo. PLoS One 8:e56144. 10.1371/journal.pone.0056144 23409142 PMC3567047

[B34] Lee A. H. Sun L. Mochizuki A. Y. Reynoso J. G. Orpilla J. Chow F.. (2021). Neoadjuvant PD-1 blockade induces T cell and cDC1 activation but fails to overcome the immunosuppressive tumor associated macrophages in recurrent glioblastoma. Nat. Commun. 12:6938. 10.1038/s41467-021-26940-2 34836966 PMC8626557

[B35] Li W. Graeber M. B. (2012). The molecular profile of microglia under the influence of glioma. Neuro Oncol. 14, 958–978. 10.1093/neuonc/nos116 22573310 PMC3408253

[B36] Liu C. Luo D. Streit W. J. Harrison J. K. (2008). CX3CL1 and CX3CR1 in the GL261 murine model of glioma: CX3CR1 deficiency does not impact tumor growth or infiltration of microglia and lymphocyt. J. Neuroimmunol. 198, 98–105. 10.1016/j.jneuroim.2008.04.016 18508133 PMC2561213

[B37] Lively S. Schlichter L. C. (2013). The microglial activation state regulates migration and roles of matrix-dissolving enzymes for invasion. J. Neuroinflammation 10:75. 10.1186/1742-2094-10-75 23786632 PMC3693964

[B38] Louis D. N. Perry A. Wesseling P. Brat D. J. Cree I. A. Figarella-Branger D.. (2021). The 2021 WHO classification of tumors of the central nervous system: a summary. Neuro Oncol. 23, 1231–1251. 10.1093/neuonc/noab106 34185076 PMC8328013

[B39] Ma D. Liu S. Lal B. Wei S. Wang S. Zhan D.. (2019). Extracellular matrix protein tenascin C increases phagocytosis mediated by CD47 loss of function in glioblastoma. Cancer Res. 79, 2697–2708. 10.1158/0008-5472.CAN-18-3125 30898840 PMC8218246

[B40] Madden K. S. Zettel M. L. Majewska A. K. Brown E. B. (2013). Brain tumor imaging: live imaging of glioma by two-photon microscopy. Cold Spring Harb. Protoc. 2013:pdb.prot073668. 10.1101/pdb.prot073668 23457348

[B41] Maezawa I. Jenkins D. P. Jin B. E. Wulff H. (2012). Microglial KCa3.1 channels as a potential therapeutic target for Alzheimer’s disease. Int. J. Alzheimers Dis. 2012:868972. 10.1155/2012/868972 22675649 PMC3364551

[B42] Maezawa I. Zimin P. I. Wulff H. Jin L. W. (2011). Amyloid-beta protein oligomer at low nanomolar concentrations activates microglia and induces microglial neurotoxicity. J. Biol. Chem. 286, 3693–3706. 10.1074/jbc.M110.135244 20971854 PMC3030372

[B44] Mieczkowski J. Kocyk M. Nauman P. Gabrusiewicz K. Sielska M. Przanowski P.. (2015). Down-regulation of IKKβ expression in glioma-infiltrating microglia/macrophages is associated with defective inflammatory/immune gene responses in glioblastoma. Oncotarget 6, 33077–33090. 10.18632/oncotarget.5310 26427514 PMC4741750

[B45] Nguyen H. M. Grössinger E. M. Horiuchi M. Davis K. W. Jin L. W. Maezawa I.. (2017). Differential Kv1.3, KCa3.1 and Kir2.1 expression in “classically” and “alternatively” activated microglia. Glia 65, 106–121. 10.1002/glia.23078 27696527 PMC5113690

[B47] Ni C. Yang L. Xu Q. Yuan H. Wang W. Xia W.. (2019). CD68- and CD163-positive tumor infiltrating macrophages in non-metastatic breast cancer: a retrospective study and meta-analysis. J. Cancer 10, 4463–4472. 10.7150/jca.33914 31528210 PMC6746141

[B49] Pellegatta S. Poliani P. L. Corno D. Menghi F. Ghielmetti F. Suarez-Merino B.. (2006). Neurospheres enriched in cancer stem-like cells are highly effective in eliciting a dendritic cell-mediated immune response against malignant gliomas. Cancer Res. 66, 10247–10252. 10.1158/0008-5472.CAN-06-2048 17079441

[B50] Qiao S. Qian Y. Xu G. Luo Q. Zhang Z. (2019). Long-term characterization of activated microglia/macrophages facilitating the development of experimental brain metastasis through intravital microscopic imaging. J. Neuroinflammation 16:4. 10.1186/s12974-018-1389-9 30616691 PMC6323850

[B51] Reich E. P. Cui L. Yang L. Pugliese-Sivo C. Golovko A. Petro M.. (2005). Blocking ion channel KCNN4 alleviates the symptoms of experimental autoimmune encephalomyelitis in mice. Eur. J. Immunol. 35, 1027–1036. 10.1002/eji.200425954 15770697

[B52] Resende F. F. Bai X. Del Bel E. A. Kirchhoff F. Scheller A. Titze-de-Almeida R. (2016). Evaluation of TgH(CX3CR1-EGFP) mice implanted with mCherry-GL261 cells as an in vivo model for morphometrical analysis of glioma-microglia interaction. BMC Cancer 16:72. 10.1186/s12885-016-2118-3 26856327 PMC4746826

[B53] Sciaccaluga M. Fioretti B. Catacuzzeno L. Pagani F. Bertollini C. Rosito M.. (2010). CXCL12-induced glioblastoma cell migration requires intermediate conductance Ca^2+^-activated K^+^ channel activity. Am. J. Physiol. Cell Physiol. 299, C175–C184. 10.1152/ajpcell.00344.2009 20392929

[B54] Sørensen M. D. Dahlrot R. H. Boldt H. B. Hansen S. Kristensen B. W. (2018). Tumour-associated microglia/macrophages predict poor prognosis in high-grade gliomas and correlate with an aggressive tumour subtype. Neuropathol. Appl. Neurobiol. 44, 185–206. 10.1111/nan.12428 28767130

[B55] Sun S. Pan X. Zhao L. Zhou J. Wang H. Sun Y. (2016). The expression and relationship of CD68-tumor-associated macrophages and microvascular density with the prognosis of patients with laryngeal squamous cell carcinoma. Clin. Exp. Otorhinolaryngol. 9, 270–277. 10.21053/ceo.2015.01305 27337949 PMC4996099

[B56] Tesileanu C. M. S. Dirven L. Wijnenga M. M. J. Koekkoek J. A. F. Vincent A. J. P. E. Dubbink H. J.. (2020). Survival of diffuse astrocytic glioma, IDH1/2 wildtype, with molecular features of glioblastoma, WHO grade IV: a confirmation of the cIMPACT-NOW criteria. Neuro Oncol. 22, 515–523. 10.1093/neuonc/noz200 31637414 PMC7158657

[B57] Toyama K. Wulff H. George Chandy K. Azam P. Raman G. Saito T.. (2008). The intermediate-conductance calcium-activated potassium channel KCa3.1 contributes to atherogenesis in mice and humans. J. Clin. Invest. 118, 3025–3037. 10.1172/JCI30836 18688283 PMC2496961

[B58] Turner K. L. Honasoge A. Robert S. M. McFerrin M. M. Sontheimer H. (2014). A proinvasive role for the Ca^2+^-activated K^+^ channel KCa3.1 in malignant glioma. Glia 62, 971–981. 10.1002/glia.22655 24585442 PMC4006152

[B59] Urbantat R. M. Jelgersma C. Brandenburg S. Nieminen-Kelhä M. Kremenetskaia I. Zollfrank J.. (2021). Tumor-associated microglia/macrophages as a predictor for survival in glioblastoma and temozolomide-induced changes in CXCR2 signaling with new resistance overcoming strategy by combination therapy. Int. J. Mol. Sci. 22:11180. 10.3390/ijms222011180 34681839 PMC8538679

[B60] Weaver A. K. Bomben V. C. Sontheimer H. (2006). Expression and function of calcium-activated potassium channels in human glioma cells. Glia 54, 223–233. 10.1002/glia.20364 16817201 PMC2562223

[B61] Wei J. Gabrusiewicz K. Heimberger A. (2013). The controversial role of microglia in malignant gliomas. Clin. Dev. Immunol. 2013:285246. 10.1155/2013/285246 23983766 PMC3741958

[B62] Weller M. van den Bent M. Tonn J. C. Stupp R. Preusser M. Cohen-Jonathan-Moyal E.. (2017). European association for neuro-oncology (EANO) guideline on the diagnosis and treatment of adult astrocytic and oligodendroglial gliomas. Lancet Oncol. 18, e315–e329. 10.1016/S1470-2045(17)30194-8 28483413

[B63] Wulff H. Miller M. J. Hansel W. Grissmer S. Cahalan M. D. Chandy K. G. (2000). Design of a potent and selective inhibitor of the intermediate-conductance Ca^2+^-activated K^+^ channel, IKCa1: a potential immunosuppressant. Proc. Natl. Acad. Sci. U S A 97, 8151–8156. 10.1073/pnas.97.14.8151 10884437 PMC16685

[B65] Zhan D. Yalcin F. Ma D. Fu Y. Wei S. Lal B.. (2022). Targeting UDP-α-d-glucose 6-dehydrogenase alters the CNS tumor immune microenvironment and inhibits glioblastoma growth. Genes Dis. 9, 717–730. 10.1016/j.gendis.2021.08.008 35782977 PMC9243400

[B67] Zhang W. Karschnia P. von Mücke-Heim I. A. Mulazzani M. Zhou X. Blobner J.. (2021). *In vivo* two-photon characterization of tumor-associated macrophages and microglia (TAM/M) and CX3CR1 during different steps of brain metastasis formation from lung cancer. Neoplasia 23, 1089–1100. 10.1016/j.neo.2021.09.001 34587566 PMC8479202

[B66] Zhang J. Li S. Liu F. Yang K. (2022). Role of CD68 in tumor immunity and prognosis prediction in pan-cancer. Sci. Rep. 12:7844. 10.1038/s41598-022-11503-2 35550532 PMC9098459

[B68] Zhu J. Zhi Q. Zhou B. P. Tao M. Liu J. Li W. (2016). The role of tumor associated macrophages in the tumor microenvironment: mechanism and functions. Anticancer Agents Med. Chem. 16, 1133–1141. 10.2174/1871520616666160520112622 27198986

