# Supplementary material for: In vivo morphological alterations of TAMs during KCa3.1 inhibition—by using in vivo two-photon time-lapse technology
Source: Front Cell Neurosci. 2022 Dec 15;16:1002487. doi: 10.3389/fncel.2022.1002487 (PMC9798303; doi:10.3389/fncel.2022.1002487)
Supplement: Supplementary file 1 [file Data_Sheet_1.pdf]

Supplementary Material

Comments on solubility of TRAM-34 and possible strategies.

We are aware that the solubility might be a limitation regarding the clinical translation, however formulations such as liposomes and nanosuspensions can be used to promote the administration to patients. Unlike lipidic or polymeric nanoparticles, which require a large amount of lipidic or polymeric excipients to encapsulate therapeutic molecules, nanosuspensions are pure forms of nanosized drug molecules stabilized by a small amount of surfactants, thereby yielding high drug loadings. To date, the application of nanosuspensions has been tested in oral, ocular and pulmonary drug delivery systems. Additionally, a recent study aimed to prepare and characterize a nanosuspension formulation of a water-insoluble isoflavone, even tested on rat glioma 2 (RG2) GBM tumor cells corroborating the hypothesis of using this administration strategy (Jacob et al., 2020; O'Mary et al., 2022; Kaplan et al., 2022, Wu et al., 2022).

Turning the treatment of GBM through the inhibition of KCa3.1 channel, Senicapoc®, a compound structurally similar to TRAM-34 has been previously tested in clinical trials on patients with sickle cell disease by oral administration, overcoming problems of solubility and administration of TRAM-34 (Ataga et al., 2009). Originally known as ICA-17043, Senicapoc®, like TRAM-34, improved in terms of both potency and selectivity for KCa3.1, blocking KCa3.1 with an IC50 of 11 nM. Senicapoc is orally bioavailable and has a half-life of 12.8 days in humans, which is significantly longer than TRAM-34 that has a half-life of 2 hours in rhesus macaques. Unfortunately, Senicapoc®, has a much shorter half-live (~1 h) in rodents making it a less optimal tool for animal studies, justifying the use of TRAM-34 on mouse model of glioblastoma (Brown et al., 2018). Moreover, considering the structural characteristic of Senicapoc® and the potential therapeutic efficacy of the inhibition of KCa3.1 channel, Senicapoc® might be an excellent substitute for the TRAM-34 in clinic.

Comparison between Figure 1D and Figure 2D/4B

We have to make several considerations to compare the Figure 1D and Figure 2D/4B and the following results: i) the preparation of the tissue; two-photon acquisition of live interaction between TAMs and glioma cells (Figure 1D) vs confocal acquisition of fixed slices in Figure 2D and 4B; ii) depth of acquisition up to 200 µm with two-photon technique in our experimental condition (Figure 1D) vs Z-stack for the confocal one (step size 0.7µm) (Figure 2D and 4B); iii) weka segmentation analysis based on positive pixel detected according to the intensity of the fluorescence signal (Figure 1D), vs counting of number of TAMs positive cells in Figure 2D and CD68 positive voxel/ total voxel in Figure 4B since CD68 has a point-like signal, iv) higher magnification (40X oil immersion objective) allowed by confocal acquisition vs 10X water immersion objective with two-photon microscope.

Taking into account the considerations reported above, we can conclude that even though the two-photon technique has higher sensitivity, higher depth of acquisition and reduced photobleaching compared to fluorescence microscopy, the following analysis based on the fluorescence intensity

might has a lower sensitivity in relation to the counting of positive cells and CD68 point-like voxel, following the confocal acquisition, as well as the different magnification can give some variability. In fact, although the result reported in Figure 1D did show no significant difference in the analysis of TAMs positive cells upon TRAM-34 treatment compared to the control condition, we can observe a decreasing trend even after 5 days of treatment, compared to the baseline.

Also, as previously reported, TAMs acquire a different morphology as function of the tumor area where they are identified; indeed, a ramified morphology was observed in the periphery of the tumor, whereas TAMs into the central region of the tumor were mostly ameboid according to various functional states that reflect the tissue environment and might influence the responsiveness to the treatment (Kvisten et al., 2019).

Additionally, in our previous work we have observed a significant higher expression of KCa3.1 (kcnn4 gene) in CD11b+ cells isolated from the ipsilateral cerebral hemisphere of glioma-bearing mice in comparison with the contralateral hemisphere and this difference was totally abolished by TRAM-34 treatment. Consistently, when CD11b+ cells isolated from human GBM specimens were treated with TRAM-34 for 24 h, a significant reduction of KCNN4 expression was observed. Similar results were obtained on cultured microglia upon glioblastoma conditioned medium (GCM) and IL-4 treatment, and KCa3.1 inhibition abolished the effect (Grimaldi et al., 2016). This evidence may suggest a higher expression of kCa3.1 channels within the tumor core, than the surrounding region, which correlates with the higher cell density (glioma and TAMs) and consequently the greater response to treatment due to an increased presence of channels. This was also confirmed by the increased expression of KCa3.1 channel in glioma patients (Turner et al., 2014) and our previous work in which we demonstrated that blockade of the calcium-activated potassium channel (KCa3.1) with TRAM-34 alone or with TMZ, reduced GL261 glioma cell migration, invasion and colony forming activity, increasing apoptosis (D'Alessandro et al., 2016), thus confirming the reduction of Ki67-positive cells within the tumor core, in line with the effect on TAMs.

So, we might speculate about the effect of TRAM-34 on both TAMs and glioma within the tumor core as it correlates with the increased cell density, the morphology of TAMs reflecting previously demonstrated phenotype of cells extracted from glioma-bearing mice (Grimaldi et al., 2016), the overexpression of the channel to which the treatment is directed.

Two-photon acquisition and analysis of TAMs

The morphometric analysis of TAMs in the tumor environment by two-photon microscopy was proposed by Resende and colleagues in a descriptive study to demonstrate the kinetic of TAMs interaction with glioma (Resende et al., 2016). The employment of Cx3cr1GFP/WT mice allowed the authors to track TAMs migration toward the tumor mass and examine the changes in cell morphology. The limitation of Cx3cr1GFP/WT mice concerns the expression of the fractalkine receptor Cx3cr1 in both brain resident microglia and bone marrow derived macrophages that cannot be discriminated against in this animal model. However, both cell populations belong to TAMs recruited into the tumor microenvironment.

The two-photon acquisition allowed as to move from the surface of the skull to a depth of 200µm. XYZ stack images were imported into Image J software for a 3D reconstruction by using the maximum intensity projection function. To follow, the Weka Segmentation plugin was used to produce a pixel-based segmentations of 3D images acquired in the tumor core, in which each pixel

was classified as belonging to GFP-positive of RFP-positive class. In this way, we quantified the % of pixel GFP-positive or RFP-positive and estimate the distribution of TAM and glioma cells in the tumor core. This live, longitudinal approach allowed us to follow the effect of TRAM-34 on tumor growth by monitoring the same animals at defined time points reducing the variability and the number of animals used for the study. At the end point of the experiment, through immunofluorescence analysis, we confirmed the reduction of TAMs density inside the tumor area, suggesting an effect of TRAM-34 on TAMs recruitment in the TME.

Images acquired via two-photon microscopy were imported into IMARIS for 3D reconstruction and the following morphology analysis was performed by using the IMARIS Surface function to estimate cell shape and size. The IMARIS Filament function was used to define the complexity of cells' ramification in terms of number of branches (ramifications), terminal points and length of ramifications, as well as cell shape and size (Chen et al, 2019).

TAMs migration analysis

XYZT time lapse images from two-photon acquisition were imported into ImageJ software using the maximum intensity projection function. The migration analysis was performed using the Image J plug in TrackMate following GFP-positive cells into the tumor microenvironment selected according to threshold and sizes and found at least in three time lapses.

1 Mice weight

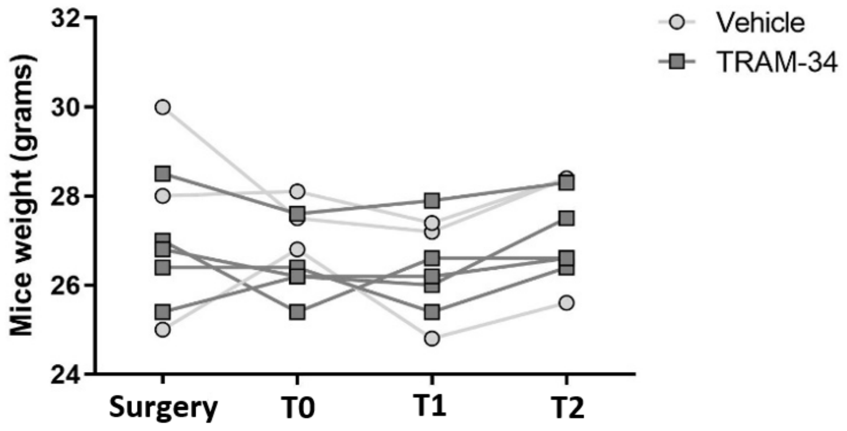

Mice weight (grams)				
	Surgery	T0	T1	T2
Vehicle	27,28±1,18	26,95±0,67	26,50±0,68	27,25±0,8
TRAM-34	26,78±0,75	26,60±0,39	26,38±0,62	27,20±0,51

Figure S1. Mice weight. The weight was checked on the day of surgery and before we started the drug administration. We then checked the weight halfway through treatment and at the end to constantly monitor animal health during the experiment. Data are the average of 4 mice per group ±s.e.

2 Morphology of TAMs

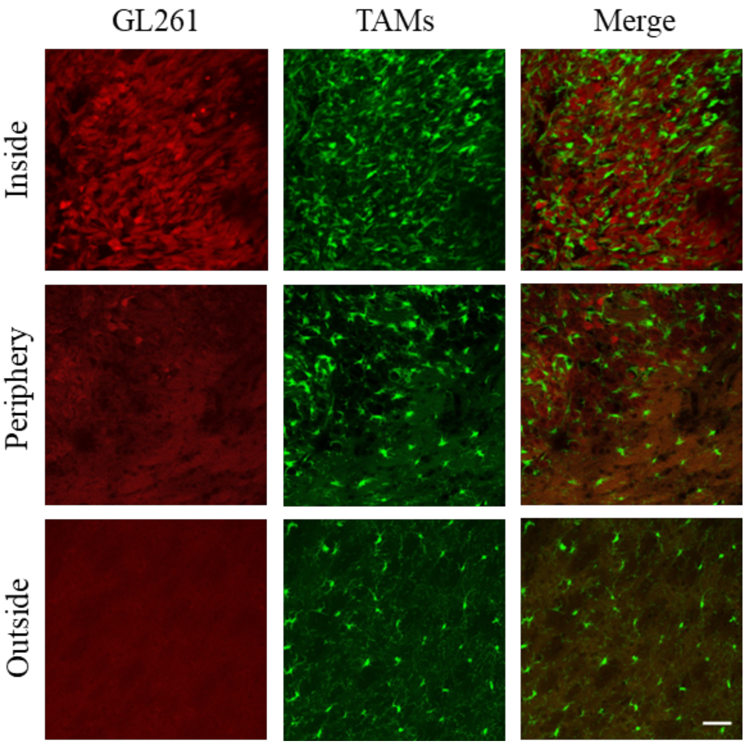

Figure S2. Morphology of TAMs. Representative confocal images of morphological changes of TAMs (green) in tumor's core, periphery and outside. The panel is composed by confocal images at 40X magnification of fixed brain slice of a not treated mice (T2). Glioma cells are labeled in red. Scale bar = 30 µm.

3 Morphology of TAMs

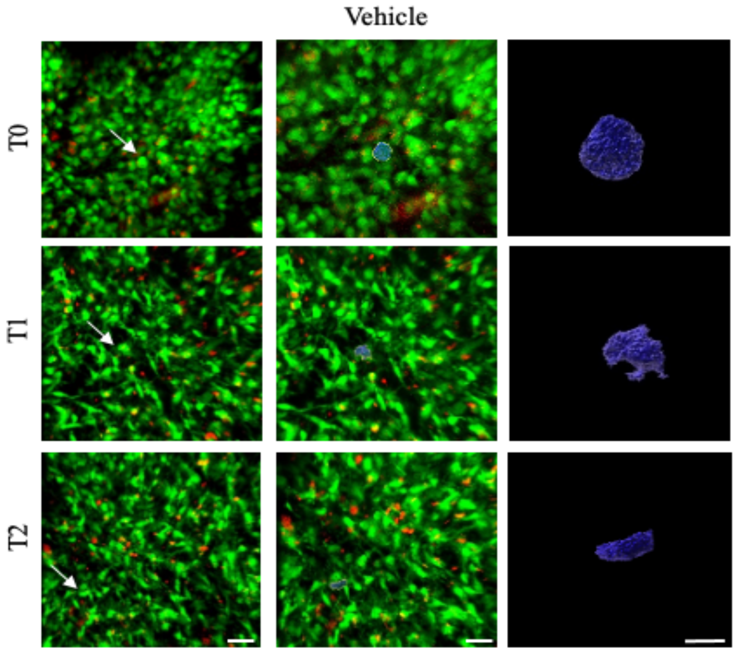

Figure S3. Morphology analysis of TAMs. (A) Representative 3D IMARIS reconstruction of TAMs. The reconstruction above was performed at T0, T1 and T2 of Vehicle treatment. The images on the center (scale bar = 20µm) and in the right (scale bar = 10µm) show the reconstruction of TAMs indicated with a white arrow in the picture on the left (scale bar = 30µm).

4 Migration of TAMs

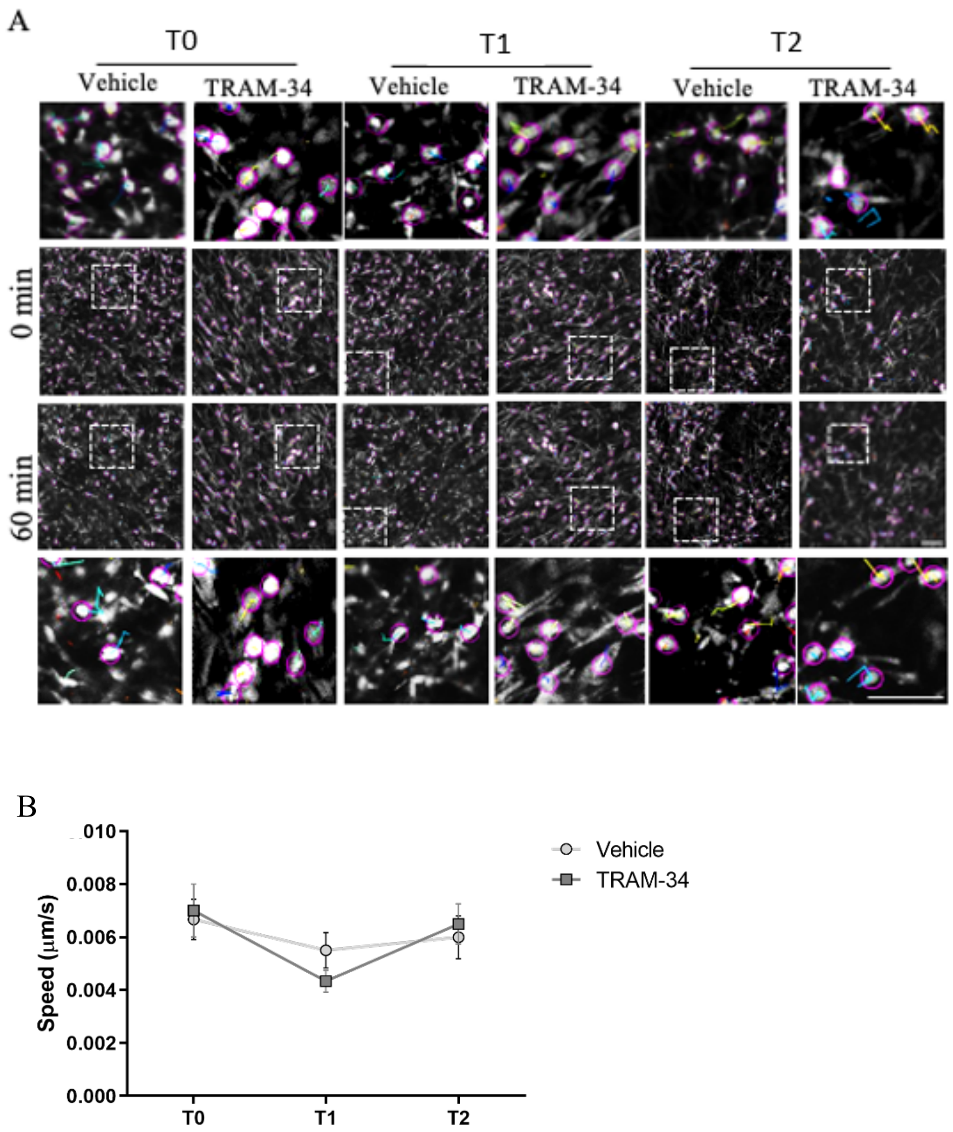

Figure S4. TAMs migration analysis in time lapse images. (A) Representative time lapse images and higher magnification of TAMs over 60 min at T0, T1, T2 of treatment with TRAM-34 (120 mg/Kg) or vehicle (100µl of peanut oil). Circles indicate individual cell position at each time point. Colored lines indicate the trajectory of the cells over the time. Scale bar = 50µm. (B) TrackMate (ImageJ) quantification of TAMs speed (µm/s) within a picture's ROI at each day of acquisition (T0, T1, T2) for vehicle-treated and TRAM-34-treated mice. The graph represents the average speed ± SEM of roughly 500 cells from 6 mice.

References

Ataga, K.I., Stocker, J. (2009) Senicapoc (ICA-17043): A potential therapy for the prevention and treatment of hemolysis-associated complications in sickle cell anemia. Expert Opin. Investig. Drugs, 18(2):231-9.

Brown, B.M., Pressley, B., Wulff, H. (2018) KCa3.1 Channel Modulators as Potential Therapeutic Compounds for Glioblastoma. Current Neuropharmacology, 16: 618-62.

Chen Z, Ross JL, Hambardzumyan D: Intravital 2-photon imaging reveals distinct morphology and infiltrative properties of glioblastoma-associated macrophages. Proc Natl Acad Sci U S A 2019;116:14254-14259.

D'Alessandro, G., Catalano, M., Sciaccaluga, M., Chece, G., Cipriani, R., Rosito, M., Grimaldi, A., Lauro, C., Cantore, G., Santoro, A., Fioretti, B., Franciolini, F., Wulff, H. & Limatola, C. (2013) KCa3.1 channels are involved in the infiltrative behavior of glioblastoma in vivo. Cell Death Dis, 4, e773.

D'Alessandro, G., Grimaldi, A., Chece, G., Porzia, A., Esposito, V., Santoro, A., Salvati, M., Mainiero, F., Ragozzino, D., Di Angelantonio, S., Wulff, H., Catalano, M. & Limatola, C. (2016) KCa3.1 channel inhibition sensitizes malignant gliomas to temozolomide treatment. Oncotarget, 7(21), 30781-96.

Grimaldi, A., D'Alessandro, G., Golia, M. T., Grössinger, E. M., Di Angelantonio, S., Ragozzino, D., Santoro, A., Esposito, V., Wulff, H., Catalano, M. & Limatola, C. (2016) KCa3.1 inhibition switches the phenotype of glioma-infiltrating microglia/macrophages. Cell Death Dis, 7, e2174.

Jacob, S., Nair, A.B., Shah, J. (2020) Emerging role of nanosuspensions in drug delivery systems. Biomater Res, 24:3.

Kaplan, A.B.U., Öztürk, H. , Çetin, M. , Vural, I., Özer, T.O. (2022) The Nanosuspension Formulations of Daidzein: Preparation and In Vitro Characterization. Turk J Pharm Sci, 19(1):84-92.

Kvisten, M., Mikkelsen, V. E., Stensjøen, A. L., Solheim, O., Van Der Want, J., Torp, S. H. (2019) Microglia and macrophages in human glioblastomas: A morphological and immunohistochemical study. Mol Clin Oncol, 11(1), 31-36.

O'Mary, H.L., Cui, Z. (2022). Injectable Formulations of Poorly Water-Soluble Drugs. In: Williams III, R.O., Davis Jr., D.A., Miller, D.A. (eds) Formulating Poorly Water Soluble Drugs. AAPS Advances in the Pharmaceutical Sciences Series, vol 50. Springer, Cham.

Resende, F. F., Bai, X., Del Bel, E. A., Kirchhoff, F., Scheller, A. & Titze-de-Almeida, R. (2016) Evaluation of TgH(CX3CR1-EGFP) mice implanted with mCherry-GL261 cells as an in vivo model for morphometrical analysis of glioma-microglia interaction. BMC Cancer, 16, 72.

Toyama, K., Wulff, H., George Chandy K., Azam P., Raman G., Saito T., Fujiwara Y., Mattson D.L., Das S., Melvin J.E., Pratt P.F., Hatoum O.A., Gutterman D.D., Harder D.R., Miura H. (2008) The intermediate-conductance calcium-activated potassium channel KCa3.1 contributes to atherogenesis in mice and humans. J Clin Invest, 118(9):3025-37.

Turner, K. L., Honasoge, A., Robert, S. M., McFerrin, M. M. & Sontheimer, H. (2014) A proinvasive role for the Ca(2+) -activated K(+) channel KCa3.1 in malignant glioma. Glia, 62(6), 971-81.

Wu, Y., Vora, L. K., Mishara, D., Adrianto, M. F., Gade, S., Paredes, A. J., Donnelly, R. F., Singh, T. R. R. (2022). Nanosuspension-loaded dissolving bilayer microneedles for hydrophobic drug delivery to the posterior segment of the eye. Biomater Adv, 137, 212767.
